# P-963. From Policy to Practice: Impact of Antimicrobial Stewardship Rounds on Rational Antimicrobial Prescribing – Experience from a Tertiary Care Centre in South India

**DOI:** 10.1093/ofid/ofaf695.1164

**Published:** 2026-01-11

**Authors:** Kavitha Jayaram, Padmaja Durga, Sukanya Sudhaharan, M V S Subbalaxmi, Kundakarla Bhanu Prasad, Roopali Somani, Sangireddy SaiSree

**Affiliations:** Nizams Institute of Medical Sciences, Hyderabad, Telangana, India; Nizams Institute of Medical Sciences, Hyderabad, Telangana, India; Nizams Institute of Medical Sciences, Hyderabad, Telangana, India; Nizams Institute of Medical Sciences, Hyderabad, Telangana, India; Nizams Institute of Medical Sciences, Hyderabad, Telangana, India; Nizams Institute of Medical Sciences, Hyderabad, Telangana, India; Nizams Institute of Medical Sciences, Hyderabad, Telangana, India

## Abstract

**Background:**

Antimicrobial resistance (AMR) is driven largely by inappropriate antibiotic use. In resource-constrained settings, structured Antimicrobial Stewardship (AMS) rounds led by a multidisciplinary team can optimize antimicrobial use and reduce AMR. We report the impact of AMS rounds at a tertiary care hospital in South India.

**Figure:**
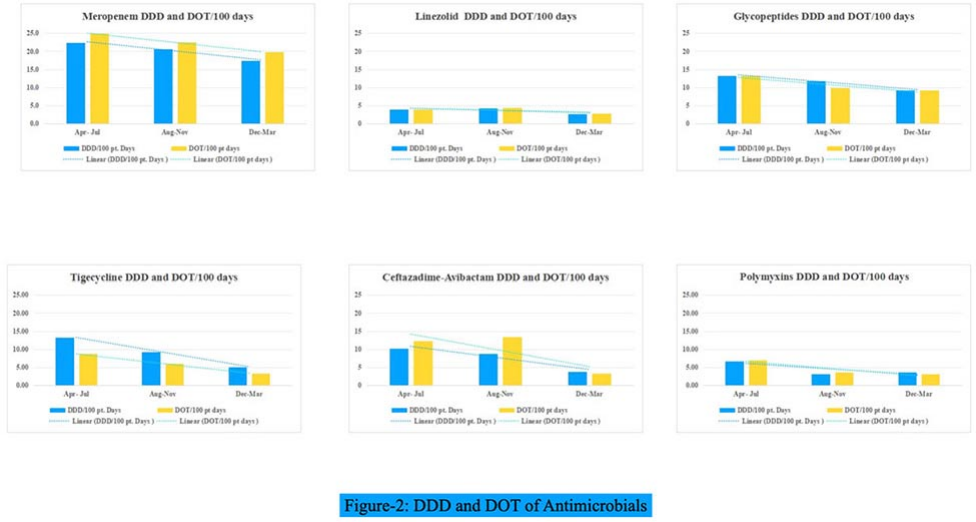
DDD and DOT of Antimicrobials

**Figure:**
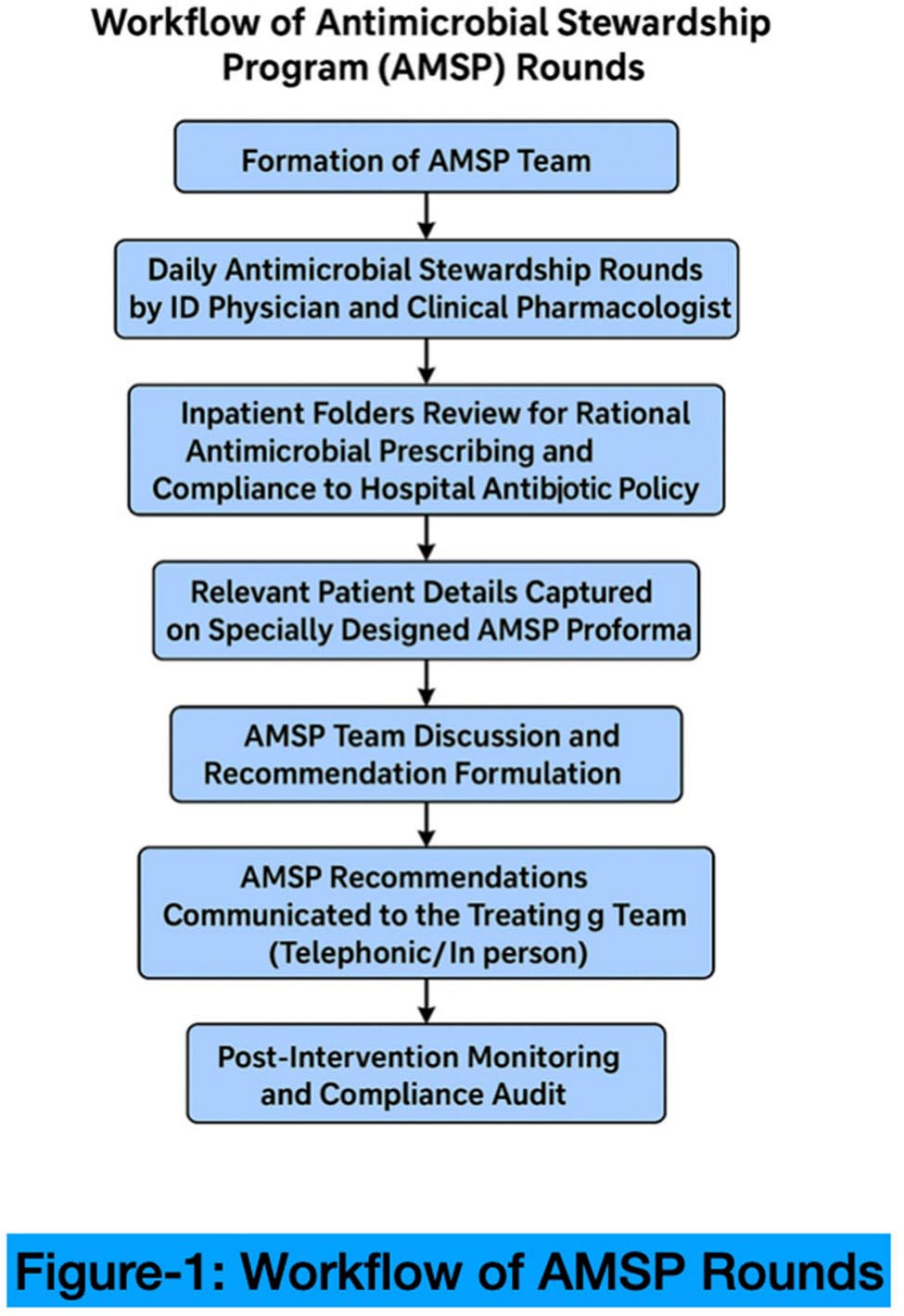
AMSP Rounds Work Flow

**Methods:**

This prospective study was conducted from April 1, 2024, to March 31, 2025, under the Indian Council of Medical Research (ICMR) AMR initiative at a tertiary care hospital. As part of the multidisciplinary AMS team, Infectious disease physicians, clinical pharmacologists and infection control nurses conducted daily stewardship rounds across 150 beds in medical and surgical ICU’s and wards. Antimicrobial prescriptions were reviewed, and structured feedback was provided to the treating teams. Process indicators such as Defined Daily Dose (DDD)/100 patient days, Days of Therapy (DOT)/100 patient days, antimicrobial consumption based on WHO AWaRe classification, culture appropriateness, and adherence to the hospital antibiotic policy were analyzed over time.

**Figure:**
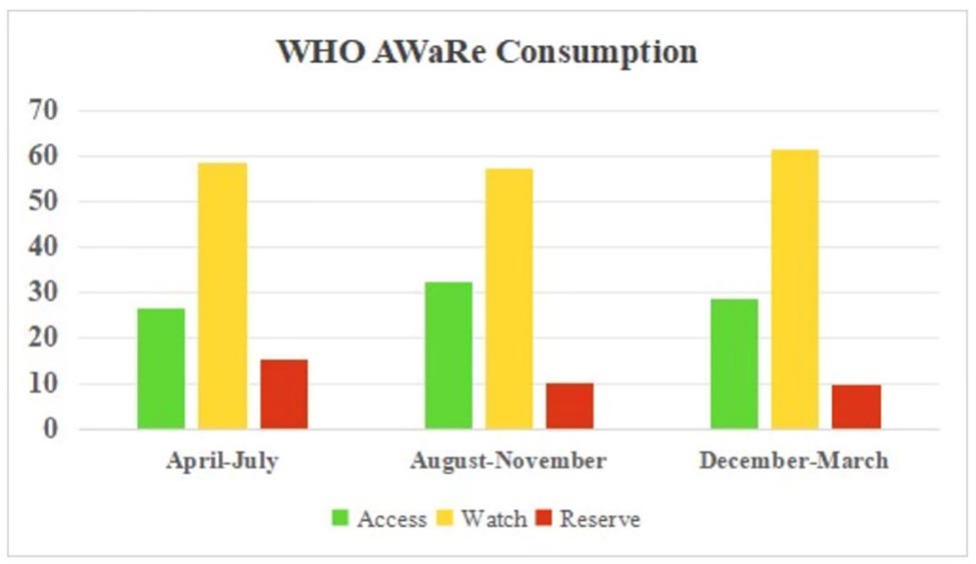
WHO Aware Classification

**Results:**

Out of 5636 patients included, 2541(45%) were admitted in ICU’s and 3095(55%) in wards. The mean DDT and DOT/100 patient days of Polymyxins, Glycopeptides, Ceftazadime-avibactam declined from April-July quarter to December-March quarter as shown in Figure 2. Watch group antibiotics accounted for highest proportion of use across all periods-58.6%, 57.4%, and 61.4% respectively. Importantly, the use of Reserve group antibiotics demonstrated a progressive decline, dropping from 15.1% in the first quarter to 10.2% and 9.9% in the subsequent quarters.

**Conclusion:**

Structured AMS rounds led to significant improvements in antibiotic prescribing, with reduced use of Reserve antibiotics and improved adherence to institutional guidelines. This model offers an effective and feasible approach for promoting rational antimicrobial use and combating AMR in resource-limited settings.

**Disclosures:**

All Authors: No reported disclosures

